# UNICORNS: Uveitis in childhood prospective national cohort study protocol

**DOI:** 10.12688/f1000research.26689.1

**Published:** 2020-10-05

**Authors:** Salomey Kellett, Jugnoo S Rahi, Andrew D. Dick, Rachel Knowles, Valerija Tadić, Ameenat Lola Solebo

**Affiliations:** 1 National Institute for Health Research Biomedical Research Centre at UCL Great Ormond Street Institute of Child Health and Great Ormond Street Hospital, London, WC1N 1EH, UK; 2 National Institute for Health Research Biomedical Research Centre at Moorfields Eye Hospital NHS Foundation Trust and UCL Institute of Ophthalmology, London, EC1V 9EL, UK; 3 Ulverscroft Vision Research Group, Institute of Child Health, University College London, London, WC1N 1EH, UK; 4 Great Ormond Street Hospital for Children NHS Trust, London, WC1N 3JH, UK; 5 Translational Health Sciences, Faculty of Health Sciences, University of Bristol, Bristol, BS8 1QU, UK; 6 Bristol Eye Hospital, University Hospitals Bristol NHS Foundation Trust, Bristol, BS1 2LX, UK; 7 School of Human Sciences, University of Greenwich, Greenwich, London, SE10 9LS, UK

**Keywords:** Uveitis, Child, Prospective Cohort, Vision, Quality of Life

## Abstract

**Background**: Childhood uveitis is a rare inflammatory eye disease which is typically chronic, relapsing-remitting in nature, with an uncertain aetiology (idiopathic). Visual loss occurs due to structural damage caused by uncontrolled inflammation. Understanding of the determinants of long term outcome is lacking, including the predictors of therapeutic response or how to define disease control.

**Aims**: To describe disease natural history and outcomes amongst a nationally representative group of children with non-infectious uveitis, describe the impact of disease course on quality of life for both child and family, and identify determinants of adverse visual, structural and developmental outcomes.

**Methods**: UNICORNS is a prospective longitudinal multicentre cohort study of children newly diagnosed with uveitis about whom a core minimum clinical dataset will be collected systematically. Participants and their families will also complete patient-reported outcome measures annually from recruitment. The association of patient (child- and treatment- dependent) characteristics with outcome will be investigated using logistic and ordinal regression models which incorporate adjustment for within-child correspondence between eyes for those with bilateral disease and repeated outcomes measurement.

**Discussion:** Through this population based, prospective longitudinal study of childhood uveitis, we will describe the characteristics of childhood onset disease. Early (1–2 years following diagnosis) outcomes will be described in the first instance, and through the creation of a national inception cohort, longer term studies will be enabled of outcome for affected children and families.

## Background

Uveitis is a collective term for a varied group of rare conditions characterised by intraocular inflammation, in which uncontrolled disease can lead to irreversible ocular damage and visual loss ^1– 3^. It is less commonly seen in children than adults, with an estimated incidence of 5/100,000 children per annum ^2, 4, 5^. Disease of childhood onset is typically chronic and relapsing-remitting in nature, and often complicated by the co-occurrence of systemic inflammatory disease ^2, 6, 7^. The primary intraocular location of the inflammation is used to classify uveitis type into anterior, intermediate, posterior and pan-(global)-uveitis ^8^. The majority of affected children (between 40–90% dependent on the studied population) have chronic anterior disease ^2, 9, 10^. Whilst up to half of childhood uveitis occurs as isolated ocular disease, uveitis can also be classified by the presence of an associated disorder ^8^, the most common being juvenile idiopathic arthritis (JIA, a group of diseases with an estimated pooled prevalence of 30 per 100,000) ^11^. Infectious causes are also well recognised but uncommon ^2^.

Affected children are managed with topical corticosteroids and systemic immunotherapies. Disease control is achieved in between 30–70% by one year after diagnosis ^1– 3, 6, 7, 9, 12^. There are uncertainties around the long-term outcomes for children with uveitis, and the determinants of those outcomes. Several relatively large but methodologically heterogenous retrospective studies have reported conflicting findings on postulated predictors of ocular outcome or therapeutic response, including gender ^12– 15^, age at onset, disease duration, or serological markers such as anti-nuclear antibody (ANA) ^1, 16– 20^. There are conflicting reports with regards to the beneficial or harmful role of topical steroids ^20, 21^, and the prognostic significance of low grade inflammation ^22, 23^. Consequently, there exists significant diversity of practice and absence of consensus on the management of disease ^24^.

In order to improve outcomes for children with non-infectious uveitis, we need to describe long term outcomes and their predictors. Existing work on the predictors of arthropathy outcomes for children with JIA suggests that these predictors may be clinical, demographic, environmental, psychological and or social ^25– 29^. We shall undertake a multi-centre, prospective inception cohort study, which aims to (1) describe disease natural history and outcomes amongst a nationally representative group of children with all-cause non-infectious uveitis, (2) describe the impact of disease course on quality of life for both child and family, and (3) identify determinants of therapeutic response, adverse visual, structural and developmental outcomes, and quality of life outcomes. We present the protocol for this study, the ‘Uveitis in Childhood Prospective National Cohort Study’, or ‘UNICORNS’.

## Methods

### Study setting

UNICORNS is a multicentre study which includes 26 secondary or tertiary care centres in the United Kingdom (UK) (see extended data - S1 ^30^), although it will also be open to any hospital based paediatric ophthalmology service delivering care to children newly diagnosed with uveitis. Whilst participant identification and data sharing will occur across all sites, the primary research centre for the study will be the UCL Great Ormond Street Institute of Child Health.

### Study population

Eligible children will be those aged under 18 years old (at diagnosis) who are newly diagnosed with uveitis. Exclusion criteria for the study includes (1) uveitis due to malignancy, ocular trauma (including iatrogenic, ie intraocular surgery), or due to confirmed ocular infection. 

### Recruitment

The families of eligible participants will be approached by their clinical team during the delivery of their routine care and informed of the study, and given contact details for the research team. Families will then contact the research team by email or by telephone to confirm their interest. A study video and study website have been established to support informed recruitment. Families will also contact researchers in person at sites where the research team are also members of clinical team. Families may also contact the research team electronically following notification of study via existing patient support groups which have agreed to support recruitment for this study (Olivia’s Vision, the largest uveitis patient group internationally). Following contact of the research team by the family, a study pack will be sent for completion. The study pack contains a participant information sheet, consent form, assent for young people, and a family background questionnaire which collects data on self-described ethnic background and the socioeconomic markers such as home ownership and parental educational attainment (see extended data - S2 ^30^). On receipt of a completed consent form, the child’s clinical team and GP will be notified of the child’s recruitment.

### Study procedures

Patient reported outcome measures (PROMs) will be collected from families at study entry and then annually over the duration of the study. The PROMs to be completed **at baseline** comprise:

- *Strengths and Difficulties Questionnaire (SDQ):* This is to screen the emotional and behavioural aspects of the participants lives. It comprises of 25 items split across 5 scales assessing emotional symptoms, conduct problems, hyperactivity-inattention, peer problems and prosocial behaviour ^31^. It takes approximately 5–10 mins to complete, and will be completed by children aged over 10 years, and by parents for children aged over 2 years. 

- *Child Health Utility 9D (CHU9D):* This is a brief (<5 mins to complete) generic preference based measure of health related quality of life, specifically developed with and for young people ^32^. It is to be completed by children aged over 7 years and parents for those aged over 2 years.

*- Children's Sleep Habits Questionnaire (CSHQ):* This evaluates the incidence of behaviours linked with typical paediatric sleep difficulties ^33^, and takes an estimated 5 to 10 mins to complete. It will be completed by children aged over 10 years, and by parents for children aged over 2 years.

*- 100-mm general evaluation (GE):* This visual analogue score (a horizontal line 100mm long) will be used to capture overall perception of the burden of the child’s uveitis. It will be completed by children aged over 5 years, and by parents for children aged 2–17.

**At one year after diagnosis, and annually**, children and their families will be invited to complete and return the following PROMS:

*- 100-mm general evaluation (GE)*

- *Child Health Utility 9D (CHU9D)*

- *Paediatric Quality of life Score (PedsQL):* This is used to evaluate health related quality of life in children. It is a brief questionnaire (typically 5 mins to complete) to be completed by children aged over 5 years, and parents of children aged over 2 years.

- *Vision related quality of life metric and Functional Vision Questionnaires for Children and Young People (VQoL_CYP & FVQ_ CYP):* This pair of instruments captures the functional and broader impacts of living with a visual disability ^24, 34^. They are self-completed for children over 8 years, and take 15 minutes to complete.

Interim analyses will be undertaken to understand the correlation between these PROMs, and in the event of strong correlation indicative of instrument redundancy, the use of the PROMS will be rationalised during the study in order to limit the time spent by the families in completing them.

At baseline all families will also be asked to complete a family background form (approx. time for completion 5 minutes). Parents of all children aged under 10 years will be asked (over the phone, or in person, approx. time for completion 5 – 10 minutes) for information available in pages of their child’s personal child health record (PCHR, or red book, current version in use since 2009). This information comprises perinatal adverse events, birth weight, vaccination history and any developmental concerns. Consent will also be sought from participating families and young people for future linkage of cohort data to routine health, social care and education databases for collection of long term broad developmental and health outcomes.

All participating centres have agreed in principle to collect, for all children presenting with uveitis, and then at every clinic appointment, a standardised clinical dataset comprising the core clinical and outcome variables (CCOV) (see extended data - S3 ^30^). These comprise ocular and systemic clinical findings at presentation, serological and imaging investigations undertaken, treatments prescribed to the children and degree of therapeutic response, and clinical outcomes such as visual acuity of the development of a sight threatening complication. This will enable exploration of confounders of disease outcome such as severity of disease at onset, age at onset, disease duration, and use of immunosuppression. Harmonisation work has been undertaken across sites ahead of study, to reach consensus on CCOV and the clinical definitions in use. The CCOV will be incorporated into routine clinical notes in either paper or electronic format, thus avoiding additional administrative burden to the collaborating clinician. Data are to be returned via postal and/or electronic transfer, or direct from clinical records by the research team. Data will be pseudonymised (through use of an alphanumeric link code) prior to return. Acquired images of the affected eyes will also, where possible, be returned to the research team via secure NHS to NHS DICOM® (Digital Imaging and Communications in Medicine) image sharing networks. Annual case note review by researchers at large volume centres (>5 children recruited per year) will occur to ensure completeness of data capture.

### Sample size

Our aim is to study all eligible children diagnosed across this multicentre network, over a period of 3 years, with an expectant consent rate of 60% and expectant attrition rate of 20%. This should result in a minimum of 250 children recruited over a three year period. This anticipated sample will allow us to reliably detect clinically important associations: at α=0.05, this sample size should >80% power to compare differences in proportions of at least 20% between groups. This assumes that the smaller group has at least 100 subjects ^35^.

### Outcome measures

These endpoints will be used, as measured every year following diagnosis:

Primary endpoints:

- New incidence of sight threatening ocular complications, including glaucoma, cataract, and macular oedema
- Quality of life

Secondary endpoints:

- Total prescribed topical and systemic corticosteroid burden
- Attainment of disease control (absolute control defined as absence of inflammation, relative control defined as the absence of inflammation greater than 0.5, the lowest grade of inflammation, with the use of less than one drop of topical corticosteroid)

The timeline for when these will be collected is provided in Table 1.

**Table 1.** Timeline showing collection of patient reported outcome measures on recruitment to UNICORN.

Procedure	Study Period		
	Baseline	Year 1	Annually
Family background questionnaire	x		
General health questionnaire	x		
SDQ	x		
CHU9D	x	x	(x)
CHSQ	x		
GE	x	x	x
VQoL_CYP / FVQ_CYP		x	(x)
PedsQL		x	(x)

*SDQ: strength and difficulties questionnaire; CHU9D: Child Health Utility 9D; CSHQ: Children's Sleep Habits Questionnaire; GE: 100-mm General Evaluation; PedsQL: Paediatric Quality of life Score; VQoL_CYP: Vision related quality of life metric – Children and Young People.* *(x): in the event of strong correlation indicative of instrument redundancy, the use of these PROMS will be rationalised during the study in order to limit the time spent by the families in completing them.*

### Statistical analysis

Patient demographic, socioeconomic and clinic characteristics, and outcomes, will be described, as will the use of and timing of the different topical and systemic agents. Continuous variables will be reported as means with standard deviations or medians with interquartile range. Categorical variables will be reported as proportions, with 95% confidence intervals. Outcomes will also be stratified by uveitis type (anterior versus other uveitis, and JIA associated versus other uveitis), by age of onset, and by use of systemic therapy within the first six months following diagnosis.

The association of patient (child- and treatment- dependent) characteristics with outcome for the largest population (those with chronic anterior uveitis, expected to be 80% of the total group), will be investigated using logistic and ordinal regression models ( STATA / R software) which incorporate adjustment for time since disease onset. Multi-level modelling will be used for adjustment for within child correspondence between eyes for those with bilateral disease, and the clustered nature of repeated measures for this chronic disease.

Functional principal component analysis (FPCA) for sparse longitudinal data will be undertaken to investigate the different trajectories of ocular inflammation amongst children with chronic anterior uveitis. The covariates used in this investigation will be those identified as potential mediators through the regression analyses. Where children have bilateral disease, the most severely affected eye will be selected for use in modelling. For those children with symmetrical bilateral disease, one eye will be chosen at random for inclusion. 

We shall also investigate the clustering of clinical findings within subtypes of all forms of uveitis disease using latent cluster analysis of demographic variables and clinical variables.

### Ethics and dissemination

The study has been approved by the Health Research Authority Research Ethics Committee (IRAS 258638, REC 20/LO/0661). This study will be registered with ClinicalTrials.org.

The results from this study will be published on the institutional website and published in peer reviewed journals. Study reports will be in accordance with the Strengthening the

Reporting of Observational Studies in Epidemiology Statement Guidelines (STROBE). Study newsletters will be made available online for participants and distributed through patient support groups. A study ‘Open Day’ will be held at the end of year 3 to inform participants on study progress and invite input on planned dissemination processes, with an online link to videos created during the day to involve those unable to attend on the day.

### Patient and public involvement (PPI)

The study aims were informed by the priorities identified by stakeholders (patients and professional groups) who participated in the 2013 James Lind Alliance Priority Setting Partnership (JLA PSP) ^36^. Amongst the top research topic priorities for those affected with inflammatory eye disease were the effectiveness of treatments, the ability to predict disease severity, the development of early detection methods, and the safety of current monitoring for ongoing chronic uveitis. 

This study is supported by the Childhood Uveitis Studies Steering group (SSG), which was formed in April 2019 in order to provide ‘stakeholder’ guidance for study aims, methodology, and dissemination plans. The group consists of three young people directly affected by childhood uveitis, and three parents of affected children. This group have been supported and trained in research methods through written materials / presentations. At least two SSG meetings have been held each year since group formation with email communications between meetings. This group has co-developed the cohort study methods – e.g. selection of patient reported outcomes and study participant literature. This study has also benefitted from regular communication with the UK based patient led support groups for childhood uveitis (Olivia’s Vision) and childhood arthritis (‘JIA matters / Versus Arthritis’).

### Data storage and management.

Data are to be entered into study specific databases and manage by the research team (SK and ALS). All information collected during the course of the study will be kept strictly confidential. Forms will be anonymised and coded using a unique patient identifier assigned at the notification stage. Stored patient information will be kept on NHS and University computers, so as to be able to track the patient in the study. All patient information will be managed according to Data Protection Act 2018 requirements.

### Data availability

The datasets generated and/or analysed during the current study will be made available (following anonymization) by the corresponding author on reasonable request. Authorised collaborators will be granted access to aggregated anonymised data from participants who have consented to this level of sharing, following review of their study protocol. Requests will be reviewed by the research team and a patient representative. Data and material transfer agreements will be required to be completed, in order to ensure regulatory compliance and that the interests of the participants are upheld and respected throughout.

Resources created through study processes will be shared upon reasonable request.

### Study status

The study is currently open to recruitment.

## Discussion

Uveitis carries the risk of blindness and can result in life long burden of avoidable disability and the attendant high financial and social costs of medical care, education, rehabilitation and support needed for visually impaired children and the adults they become. Improved understanding of the factors associated with favourable and adverse outcomes for affected children are necessary for planning care and service provision. This multicentre study will result in a nationally representative cohort of children with non-infectious uveitis, providing externally valid findings on the determinants of outcome. The prospective collection of patient reported outcome alongside an agreed minimal core clinical dataset will enable robust evaluation of the role of postulated risk factors with adjustment for identified confounding. It will also provide valuable information on the lived experience of these children, through the use of a range of patient reported measures. This allows UNICORNS to capture a more complete spectrum of patient centred outcomes than have been reported by previous studies. This study also offers a timely opportunity to investigate the outcomes of childhood uveitis in the United Kingdom at a time when more targeted, biologic agents such as adalimumab and tocilizumab have either been commissioned, or recommended for use in children with refractory disease ^37, 38^. Within trial populations, up to a quarter of children continue to have uncontrolled disease despite the use of these agents ^37^. UNICORNS will provide information on the feasibility of undertaking future ‘Trial within cohorts’ or other pragmatic interventional trial designs, and provide a data sharing infrastructure to support ‘classic’ randomised controlled trials of emerging novel interventions. Greater understanding of this population of children affected by a rare, chronic, inflammatory, idiopathic disease will also allow investigators to develop future nested trials of complex interventions which are targeted at patient (eg, packages of support around the time of transition and transfer to adult care), clinician (eg, decision support models for rare disease) or organisation level (eg, information sharing platform across primary and secondary care for rare disease). Thus, the findings of this cohort study will be of direct importance to clinical practice and future research within this disease area and beyond.

### Limitations

This study design involves additional burden on the participants and their family through the completion of multiple carefully chosen and validated patient report metrics. These metrics characterise the family experience, global and vision related quality of life and health related utility. This allows for data collection on outcomes that our PPI work has shown to be of crucial importance to the affected families.

Observational studies such as UNICORNS are open to confounding, preventing causal inference when associations are reported. Our prospective design and use of a core minimal dataset should enable careful consideration of identified potential confounders. In order to strengthen study findings, all study reports will be reported in accordance with STROBE guidelines.

Attrition is always a concern with regards to longitudinal studies. This study is supported by a national clinical network, the Paediatric Ocular Inflammation Group ^24, 39^, which will support follow up should children transfer from one clinical unit to another. The involvement of the child’s primary care giver, and the support of patient groups and their continued publication of study newsletters, is also expected to reduce attrition rates.

### Summary

Childhood onset uveitis confers particular burden due to chronicity, the association with systemic inflammatory disease, and the frequent requirement for systemic immunosuppression. There remain unanswered questions around disease phenotypes, long-term eye and wider developmental outcomes, and the determinants of those outcomes, with resultant limitations in the evidence base used to counsel affected families, balance treatment decisions, or plan further research. We propose a population based, prospective longitudinal study of childhood uveitis, in order to describe the characteristics of childhood onset disease. We will describe outcome and investigate socio-demographic, clinical, biological and treatment related determinants of outcome. Early (1–2 years following diagnosis) outcomes will be described in the first instance, and through the creation of a national inception cohort, we shall enable longer term studies of outcome for affected children and families. 

### Ethics approval and consent to participate

This study is approved by the UK Health Research Authority. Patients or their legal representative will have to sign an informed consent form before study entry. This study is in accordance with the Declaration of Helsinki and in accordance with the Medical Research Involving Human Subjects Act (WMO). Risks for participating in this study are expected to be very small to negligible and all patients will receive standard care. Objection or incapacitation of a patient or their representative will lead to exclusion from the study and analysis.

## Data availability

### Underlying data

No data are associated with this article.

### Extended data

Open Science Framework: UNICORNS extended data: https://doi.org/10.17605/OSF.IO/EUBMA ^30^

This project contains the following extended data:

- S1.pdf (List of collaborating centres)
- S2.pdf (Study information pack)
- S3.pdf (Minimal uveitis core dataset)

Data are available under the terms of the Creative Commons Zero "No rights reserved" data waiver (CC0 1.0 Public domain dedication).
