# Supplementary material for: UNICORNS: Uveitis in childhood prospective national cohort study protocol
Source: F1000Res. 2023 Aug 30;9:1196. Originally published 2020 Oct 5. [Version 2] doi: 10.12688/f1000research.26689.2 (PMC10905007; doi:10.12688/f1000research.26689.2)
Supplement: Peer review history

# Reviewer report: Reviewer response for version 2

**DOI:** 10.5256/f1000research.155261.r245148

**Published:** 2024-02-23

**Authors:** Piergiorgio Neri

**Affiliations:** 1 The Eye Institute, Cleveland Clinic Abu Dhabi, Abu Dhabi, Abu Dhabi, United Arab Emirates

1. The manuscript detailing the UNICORNS study offers a pioneering and comprehensive exploration into childhood uveitis, a rare but significant inflammatory eye condition known for its chronic, relapsing-remitting nature and idiopathic origins. This ambitious research project stands out for its meticulous approach to understanding the disease's natural history, its impact on the quality of life of the affected children and their families, and the determinants of adverse visual, structural, and developmental outcomes. The study promises to systematically gather and analyze data from children newly diagnosed with non-infectious uveitis nationwide by employing a prospective longitudinal multicentre cohort design.
2. Including patient-reported outcome measures alongside a core minimum clinical dataset is particularly commendable as it highlights the study's holistic approach, emphasizing the disease's physical, emotional, and psychological ramifications. The analytical strategy, employing logistic and ordinal regression models that account for the unique challenges posed by bilateral disease and repeated outcome measurements, showcases the research team's commitment to methodological rigor and precision.
3. This study is poised to significantly advance our understanding of childhood uveitis by identifying critical therapeutic response predictors and defining disease control criteria. The creation of a national inception cohort for childhood uveitis enables the detailed characterization of the disease's onset and early outcomes and lays the groundwork for future investigations into the long-term consequences for affected individuals and their families. Through its thoughtful design and execution, the UNICORNS study represents a critical step forward in pediatric ophthalmology research, with the potential to inform more effective management strategies and ultimately improve the lives of those impacted by this challenging condition.

Is the study design appropriate for the research question?

Yes

Is the rationale for, and objectives of, the study clearly described?

Yes

Are sufficient details of the methods provided to allow replication by others?

Yes

Are the datasets clearly presented in a useable and accessible format?

Yes

Reviewer Expertise:

Immunology, Translational Medicine, Immune-pharmacology, Pharmacovigilance, Ophthalmology, Infectious Diseases, Medical Retina, Academic Research.

I confirm that I have read this submission and believe that I have an appropriate level of expertise to confirm that it is of an acceptable scientific standard.

# Reviewer report: Reviewer response for version 1

**DOI:** 10.5256/f1000research.29466.r75910

**Published:** 2021-01-04

**Authors:** Ivan Foeldvari

**Affiliations:** 1 Hamburg Centre for Pediatric and Adolescence Rheumatology Centre for Treatment of Scleroderma and Uveitis in Childhood and Adolescence Teaching Unit of the Asklepios Campus of the Semmelweis Medical School, Schön Klinik Hamburg Eilbek Dehnhaide, Hamburg, Germany

- This is a unique project to learn more about childhood uveitis. Patients are assessed in a standardized manner regarding uveitis visual outcome and PROMs.
- I am missing the application for EYEQL ( 10.1002/acr.20524 ^1^).
- It would be a great chance to apply the MIWGUC outcome measures prospectively, which offer a definition for inactive disease and response to treatment (10.1186/s12969-019-0345-2 ^2^).
- I would suggest an assessment of the patients every 6 months, the proposed annual follow up interval is to rare.
- I missed the collection of data of activity of the associated condition, like JIA, sarcoidosis...

Is the study design appropriate for the research question?

Yes

Is the rationale for, and objectives of, the study clearly described?

Yes

Are sufficient details of the methods provided to allow replication by others?

Yes

Are the datasets clearly presented in a useable and accessible format?

Yes

Reviewer Expertise:

Pediatric Rheumatology, outcome research, juvenile idiopathic arthritis associated uveitis, juvenile localized scleroderma, juvenile systemic sclerosis

I confirm that I have read this submission and believe that I have an appropriate level of expertise to confirm that it is of an acceptable scientific standard, however I have significant reservations, as outlined above.

## References

[1] : Development of a vision-related quality of life instrument for children ages 8-18 years for use in juvenile idiopathic arthritis-associated uveitis. *Arthritis Care Res (Hoboken)* .2011;63(9) : 10.1002/acr.20524 1254-61 10.1002/acr.20524 21678564 PMC3169749

[2] : Proposal for a definition for response to treatment, inactive disease and damage for JIA associated uveitis based on the validation of a uveitis related JIA outcome measures from the Multinational Interdisciplinary Working Group for Uveitis in Childhood (MIWGUC). *Pediatr Rheumatol Online J* .2019;17(1) : 10.1186/s12969-019-0345-2 66 10.1186/s12969-019-0345-2 31575380 PMC6774210

# Response

**Published:** 2021-04-21

**Authors:** Ameenat Lola Solebo

**Affiliations:** Great Ormond Street Institute of Child Health, UK

We thank Prof Foeldvari very much for his comments. Responses to his suggestions for amendments to study methods are below: 

- I am missing the application for EYEQL ( 10.1002/acr.20524 ^1^ ).

UNICORNS will only be using metrics which have been validated for use in a UK cohort, and we look forward to using EYEQL once it has not been validated for use in a UK cohort

- I would suggest an assessment of the patients every 6 months, the proposed annual follow up interval is to rare.

Children will continue to receive routine clinical care, which involves follow up at intervals of between 1 week and 4 months. The annual interval refers only to the collection of the specific patient reported outcome metrics.  

- I missed the collection of data of activity of the associated condition, like JIA, sarcoidosis.

Data on general health status is routinely collected at clinical follow up (as indicated in the dataset documents) and will be extracted for use in UNICORNS analyses.

# Reviewer report: Reviewer response for version 1

**DOI:** 10.5256/f1000research.29466.r72432

**Published:** 2020-10-23

**Authors:** Joke de Boer

**Affiliations:** 1 Ophthalmo-Immunology Unit, University Medical Center Utrecht, Utrecht University, Utrecht, The Netherlands

This is an important research project that will provide new information to improve treatment strategies in children with non-infectious uveitis. This project will provide an unique cohort of children with uveitis that longitudinally will be followed. The questionnaires will provide important information regarding the impact on patients suffering from childhood uveitis and their families. The outcome of QqL might also influence the care of these patients. The study is very well designed and patients are involved in the design of the project. The inclusion of 250 patients in 26 centers in 3 years is highly feasible.

 The topics that need further clarification are the serology tests that are performed and how infectious causes are excluded.

 However, several questions remain regarding the secondary endpoint for attainment of disease control. The authors state that absolute disease control is defined as absence of inflammation and relative disease control as absence of inflammation greater than 0,5, the lowest grade of inflammation. These definitions should be more specified because they might be different for the different anatomic subtypes of uveitis. For instance, is flare a grade of inflammation? Flare might permanently be raised after inflammation in anterior uveitis without cells in anterior chamber. Another example is intermediate uveitis where cells might be absent in anterior chamber in active diseases, however, cell in vitreous might persist for a long period without signs of active inflammation on fluorescein angiography. So disease control should be more specified for different anatomic subtypes of uveitis. Also the inflammatory parameters in the different anatomic subtypes that will be assessed should be mentioned.

Is the study design appropriate for the research question?

Yes

Is the rationale for, and objectives of, the study clearly described?

Yes

Are sufficient details of the methods provided to allow replication by others?

Partly

Are the datasets clearly presented in a useable and accessible format?

Not applicable

Reviewer Expertise:

My area of research is uveitis in children and adults.

I confirm that I have read this submission and believe that I have an appropriate level of expertise to confirm that it is of an acceptable scientific standard.
